# Attention-guided variational graph autoencoders reveal heterogeneity in spatial transcriptomics

**DOI:** 10.1093/bib/bbae173

**Published:** 2024-04-16

**Authors:** Lixin Lei, Kaitai Han, Zijun Wang, Chaojing Shi, Zhenghui Wang, Ruoyan Dai, Zhiwei Zhang, Mengqiu Wang, Qianjin Guo

**Affiliations:** Academy of Artificial Intelligence, Beijing Institute of Petrochemical Technology, Beijing 102617, China; Academy of Artificial Intelligence, Beijing Institute of Petrochemical Technology, Beijing 102617, China; Academy of Artificial Intelligence, Beijing Institute of Petrochemical Technology, Beijing 102617, China; Academy of Artificial Intelligence, Beijing Institute of Petrochemical Technology, Beijing 102617, China; Academy of Artificial Intelligence, Beijing Institute of Petrochemical Technology, Beijing 102617, China; Academy of Artificial Intelligence, Beijing Institute of Petrochemical Technology, Beijing 102617, China; Academy of Artificial Intelligence, Beijing Institute of Petrochemical Technology, Beijing 102617, China; Academy of Artificial Intelligence, Beijing Institute of Petrochemical Technology, Beijing 102617, China; Academy of Artificial Intelligence, Beijing Institute of Petrochemical Technology, Beijing 102617, China

**Keywords:** spatially resolved transcriptomics, spatial clustering, graph deep learning, variational graph autoencoder, attention-guided

## Abstract

The latest breakthroughs in spatially resolved transcriptomics technology offer comprehensive opportunities to delve into gene expression patterns within the tissue microenvironment. However, the precise identification of spatial domains within tissues remains challenging. In this study, we introduce AttentionVGAE (AVGN), which integrates slice images, spatial information and raw gene expression while calibrating low-quality gene expression. By combining the variational graph autoencoder with multi-head attention blocks (MHA blocks), AVGN captures spatial relationships in tissue gene expression, adaptively focusing on key features and alleviating the need for prior knowledge of cluster numbers, thereby achieving superior clustering performance. Particularly, AVGN attempts to balance the model’s attention focus on local and global structures by utilizing MHA blocks, an aspect that current graph neural networks have not extensively addressed. Benchmark testing demonstrates its significant efficacy in elucidating tissue anatomy and interpreting tumor heterogeneity, indicating its potential in advancing spatial transcriptomics research and understanding complex biological phenomena.

## INTRODUCTION

In spatially resolved transcriptomics (SRT) studies [1–4], identifying spatial domains [5–8] is a critical task. Currently, techniques for delineating spatial domains can be classified into two primary categories: non-spatial approaches [9–13] and spatial clustering methods [8, 12, 14–16]. Traditional non-spatial methods primarily utilize clustering algorithms [17] such as *K*-means [18] and Louvain [19] However, their progress is limited due to technological fluctuations, resulting in either too few or overly sparse spots [20, 21]. Another approach involves defining cell type features through convolutional single-cell RNA-seq [22], but experiments demonstrate their inadequacy for achieving cell- or subcellular-level resolution in ST data. These approaches utilize gene expression data as input. However, since the cells must be scrambled to prepare the sample in the experiment, the location information of the cells is lost, and as a result, the clustering results are often not closely related to the actual structure of the tissue slice.

Therefore, considering the relationship between gene expression [23], spatial location and tissue morphology [24] becomes particularly important to better connect gene expression and spatial positional information. Some relevant studies have made attempts in this direction. For example, BayesSpace [8] adopts Bayesian methods and introduces spatial priors to encourage nearby points to belong to the same cluster. stLearn [16] utilizes morphological distance and spatially neighboring structures to standardize gene expression data. SpaGCN [25] integrates gene expression, spatial location and morphological data to discern spatial domains through the creation of an undirected weighted graph. SEDR [26] employs a deep autoencoder network to embed spatial information. DeepST [27] enhances data representation by using graph neural networks (GNNs) to construct a denoising autoencoder. The spatial-ID [28] method leverages transfer learning techniques to construct probability distributions from learned single-cell RNA sequencing (scRNA) data, forming pseudo-labels. These are then combined with a variational autoencoder for cell identity characterization on a reference dataset with clearly defined cell types. The PAST [29] method integrates self-attention mechanisms [30] with residual networks and graph convolutional networks to extract latent features from SRT data. The STAGATE [31] method incorporates graph attention mechanisms into encoder and decoder operations, interpreting spatial domains in spatial transcriptomics and showcasing functionality in three-dimensional visualization. STAGATE demonstrates the potential application capabilities of attention mechanisms in understanding the similarity between adaptively adjacent points and representing spatial domain boundaries.

Among the methods discussed, the application of GNN methods [32–39], particularly utilizing the variational graph autoencoder (VGAE), demonstrates notably superior ARI scores in benchmark testing. In methods like PAST and STAGATE, additional attention layers are incorporated to further enhance the clustering performance of the model. However, limitations persist regarding the balance between attention to local and global structures. Additionally, research on tasks involving clustering without prior knowledge of the number of clusters remains relatively limited, and the performance of traditional clustering algorithms is often impeded as they typically rely on user-provided prior information to determine the number of clusters. Lastly, effectively leveraging slice data, spatial information and raw gene expression from SRT data to construct enhanced gene expression matrices for calibrating low-quality gene expression remains a challenge.

Here, we introduce AttentionVGAE (AVGN), which leads the VGAE [39] by incorporating modules containing multi-head attention (MHA blocks) [40–42] to identify spatial domains. Specifically, AttentionVGAE utilizes pre-trained MaxVit networks to extract latent features from slice images and combines them with raw expression data and positional information of spatially adjacent points to construct an enhanced gene expression matrix. Subsequently, latent representations are obtained from the enhanced gene expression matrix using a graph convolutional encoder with MHA blocks. Finally, the reparameterization and graph deconvolution decoder are employed to reconstruct unstructured information. Notably, the introduction of MHA blocks in AttentionVGAE enables the network to concentrate attention on specific regions or features while also focusing on multiple key features in spatial data, thereby effectively capturing the complex relationships in gene expression within tissues. This facilitates learning more general and abstract feature representations, enhancing the network’s generalization capability. Additionally, AttentionVGAE attempts to adaptively adjust its focus on potential clustering structures in the data with the assistance of MHA blocks, thereby improving the flexibility and efficacy of clustering algorithms. Consequently, AttentionVGAE not only calibrates low-quality gene expression by integrating slice images, spatial dependencies and gene expression but also enhances the balance between attention to local and global structures, thus improving the performance of identifying spatial domains.

Through benchmark testing against six state-of-the-art methods on SRT datasets from 10x Visium, AttentionVGAE demonstrates higher clustering accuracy. It elucidates fine-grained anatomical regions in the adult mouse brain dataset from 10x Visium and the Slide-seq mouse hippocampus dataset. Moreover, AttentionVGAE is employed to decipher tumor heterogeneity in adult Human Glioblastoma Multiforme and infiltrative ductal carcinoma datasets [43, 44], providing deeper biological insights into cancer-related genes and serving as a valuable reference for subsequent analysis combining other clinical data [45–47].

## MATERIALS AND METHODS

### Dataset

In this manuscript, to comprehensively assess the performance of AttentionVGAE compared to state-of-the-art spatial detection methods, we utilized a highly regarded dataset of the human dorsolateral prefrontal cortex (DLPFC) [43] as a benchmark. This dataset was obtained from the 10x Visium platform and includes data from three subjects, totaling 12 sections. The anatomical coverage of each section spans all six layers of neuronal cells and the cortex, resulting in a total of seven layers.

Additionally, diverse datasets were employed, including coronal slices of the adult mouse brain (fresh-frozen). There are 2702 spots under tissue with an average of 115 569 reads per spot and a median of 6018 genes per spot.

Furthermore, data from adult human glioblastoma cells were used, comprising 3468 spots under tissue with an average of 16 383 reads per spot. Notably, 87.4% of reads confidently mapped to the targeted transcriptome. Another dataset involved invasive ductal carcinoma, with 4727 spots under tissue, averaging 40 795 reads per spot and a median of 2964 genes per spot. All these datasets originated from the 10x Visium platform.

Samples from the Stereo-seq platform were obtained, including the first four sagittal slices from the early developmental stages of C57BL/6 mouse embryos, selected from the MOSTA database [44]. These slices correspond to E9.5 (~7.1 mm^2^), E10.5 (~11.5 mm^2^), E11.5 (~18.8 mm^2^) and E12.5 (~32.1 mm^2^), chosen to capture crucial developmental milestones. Also from the MOSTA database are the Dorsal_midbrain_celand data and Adult mouse hemi-brain data, providing molecular insights into the gene expression dynamics during early mouse embryonic development.

Finally, samples from the Slide-seq platform [45], including the Hippocampus dataset, were included. This dataset contributes to a further evaluation of AttentionVGAE’s effectiveness in handling high-spatial-resolution data, demonstrating its adaptability across different platforms.

### Preprocessing

To fully leverage image information for spatial domain detection tasks, AVGN employs a pre-trained MaxVit [46] model. Initially, AVGN performs image segmentation on each data point in the spatial transcriptomics data, dividing the images into small blocks. The size of the segmented image blocks is set to 224 pixels × 224 pixels, encompassing three channels (RGB). These small image blocks serve as inputs to the MaxVit model, which has an input layer dimension of (1, 3, 224, 224), where 1 represents batch size, 3 represents the number of channels and 224x224 denotes the image dimensions. The MaxVit model extracts features from each image block, and the resulting feature matrix, MaxVit(input), has dimensions equal to the predefined number of PCA [47] principal components, typically set to 50.

To obtain an augmented gene expression matrix, the enhanced expression ($\tilde{{\mathbf{E}}_{\mathbf{a}}}$) for the gene expression (${\boldsymbol{E}}_{\boldsymbol{a}}$) at any point a in the spatial domain is calculated using the following formula:


(1) \begin{equation*} \tilde{{\mathbf{E}}_{\mathbf{a}}}={\boldsymbol{E}}_{\boldsymbol{a}}+\frac{\sum_{\boldsymbol{b}=\mathbf{1}}^{\boldsymbol{n}}{\boldsymbol{E}}_{\boldsymbol{b}}\cdotp{\boldsymbol{C}}_{\boldsymbol{a}\boldsymbol{b}}\cdotp{\boldsymbol{R}}_{\boldsymbol{a}\boldsymbol{b}}\cdotp{\boldsymbol{A}}_{\boldsymbol{a}\boldsymbol{b}}}{\boldsymbol{n}} \end{equation*}

The *b* represents the set of *n* neighbors associated with point *a* (in AVGN, the default is to calculate the radius a based on the mean and variance of the distances to the six nearest neighboring points, considering all points within a distance less than *r* as neighbors of point a). ${\boldsymbol{C}}_{\boldsymbol{ab}}$ is the cosine similarity between the gene expression profiles of points a and b, ${\boldsymbol{A}}_{\boldsymbol{ab}}$ indicates whether the two points are neighbors (equals 1 if they are neighbors, 0 otherwise) and ${\boldsymbol{R}}_{\boldsymbol{ab}}$ represents the gene expression weight between the two points, calculated as follows:


(2) \begin{equation*} {\boldsymbol{R}}_{\boldsymbol{a}\boldsymbol{b}}=\mathbf{1}-\frac{\left({\boldsymbol{E}}_{\boldsymbol{a}}-\overline{{\boldsymbol{E}}_{\boldsymbol{a}}}\right)\cdotp \left({\boldsymbol{E}}_{\boldsymbol{b}}-\overline{{\boldsymbol{E}}_{\boldsymbol{b}}}\right)}{{\left\Vert \left({\boldsymbol{E}}_{\boldsymbol{a}}-\overline{{\boldsymbol{E}}_{\boldsymbol{a}}}\right)\right\Vert}_{\mathbf{2}}\cdotp{\left\Vert \left({\boldsymbol{E}}_{\boldsymbol{b}}-\overline{{\boldsymbol{E}}_{\boldsymbol{b}}}\right)\right\Vert}_{\mathbf{2}}} \end{equation*}

AVGN constructs a KDTree [48] using enhanced gene expression data and point coordinate information. KDTree is a tree-like data structure used for efficient searching of nearest neighbors in multi-dimensional space. Utilizing the constructed KDTree, a nearest neighbor search is performed for each point in the dataset, returning the distances and indices of the nearest *k* + 1 neighbors for each point. Subsequently, by iterating over each data point, edges are added to the graph structure connecting it with its *k* nearest neighbors, and these edges are stored in a graphList. The inner loop iterates over each row corresponding to a data point in the dataset, adding the index of the current point and its *k* nearest neighbors to the graphList. This process establishes a graph in which each data point is connected to its *k* nearest neighbors.

Finally, the data are normalized [49] to ensure that the sum of gene expression values for each cell is equal to 1. Logarithmic transformation and standardization are applied to scale down the magnitude of the data and ensure that the expression values for each gene have similar scales across all cells. Principal Component Analysis (PCA) is employed to reduce the dimensionality of the data to a specified number of principal components (default set to 128 dimensions in AVGN). This series of processing steps helps eliminate differences between cells, providing more comparable data for subsequent analysis.

### Encoder components

For each element ${x}_{ij}^{\log -\mathrm{transformed}}$, the normalization operation involves the following two steps:

Calculate the mean$\left({\mu}_j\right)$ and standard deviation$\left({\sigma}_j\right)$ for each feature:


(3) \begin{equation*} {\mu}_j=\frac{1}{n}\sum_{i=1}^n\kern0.1em {x}_{ij}^{\log -\mathrm{transformed}} \end{equation*}

(4) \begin{equation*} {\sigma}_j=\sqrt{\frac{1}{n}\sum_{i=1}^n\kern0.1em {\left({x}_{ij}^{\log -\mathrm{transformed}}-{\mu}_j\right)}^2} \end{equation*}

where $n$represents the sample size.

Standardize each element ${x}_{ij}^{\log -\mathrm{transformed}}$ to obtain the standardized element ${x}_{ij}^{\mathrm{standardized}}$:


(5) \begin{equation*} {x}_{ij}^{\mathrm{standardized}}=\frac{x_{ij}^{\log -\mathrm{transformed}}-{\mu}_j}{\sigma_j} \end{equation*}

This normalization process standardizes the distribution of each data feature, giving it a mean of 0 and a variance of 1. This standardized format enhances the ease of subsequent analysis and modeling.

### Multi-head attention mechanism components

This powerful technique enhances a neural network’s ability to process data by employing parallel computations and multiple heads. This improves the model’s representational capacity and its ability to capture diverse relationships. The original input is a vector of dimensions ${d}_{\mathrm{model}}$. This input is linearly transformed by a linear layer, mapping it to $h$ different subspaces, each with a dimension ${d}_k=\frac{d_{\mathrm{model}}}{h}$ . This operation can be seen as segmenting the input features. Independent attention calculations are then performed for each subspace in parallel, improving processing efficiency. Subsequently, the information from different subspaces is fused together. Finally, the integrated attention vectors undergo another linear layer to map them back to the original ${d}_{\mathrm{model}}$ dimension. In AVGN, we introduced the MAH block, which is composed of a linear layer stacked on top of a multi-head attention layer. The objective is to allow the linear layer to learn the latent representation of input features. This is achieved by parallel computation through the multi-head attention layer, enhancing the model’s information capture capabilities. The input dimension of the linear layer is 128, with an output dimension of 32, while the input and output dimensions of the multi-head attention layer are both set to 32.


$$ hea{d}_i=\mathrm{Attention}\left(Q{W}_i^Q,K{W}_i^K,V{W}_i^V\right). $$

(6) \begin{equation*} \mathrm{MultiHead}\left(Q,K,V\right)=\mathrm{Concat}\left( hea{d}_1,\dots, hea{d}_h\right){W}^O \end{equation*}

Each attention head can learn distinct relationships, empowering the model to more effectively capture various facets of information within the input sequence. Consequently, this enhances the model’s generalization and representational capabilities.

### VGAE components

**Figure 1:**
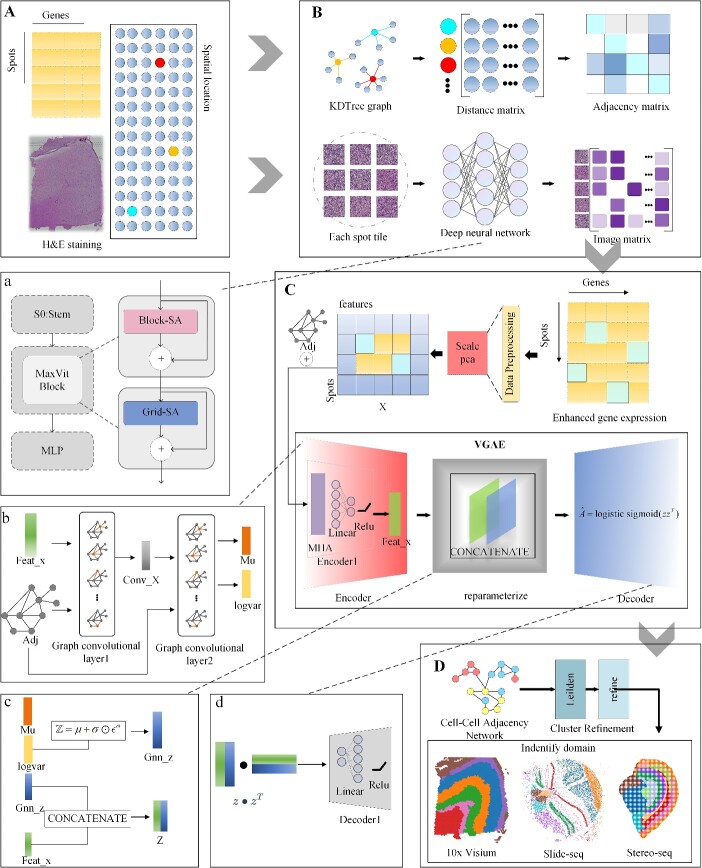
The workflow of AttentionVGAE. (**A**) Data required for the process includes hematoxylin and eosin (H&E)-stained histological images (optional), spatial gene expression, and spatial coordinates. (**B**) AttentionVGAE computes the adjacency matrix and utilizes a pre-trained MaxVit [46] to learn features present in the images, followed by appropriate normalization [47]. (**C**) The preprocessed feature matrix and adjacency matrix are input into the optimized VGAE to extract latent embeddings. (**D**) Based on the latent embeddings, a cell adjacency network is constructed for further clustering operations, and the results of spatial domain detection are visualized [50]. (**a**) Details of the MaxVit block. (**b**) Data flow display in encoder. (**c**) The calculation process in reparameterization. (**d**) Figure deconvolution decoder working details.

The main difference between VGAE and VAE lies in the use of a multidimensional Gaussian distribution to replace the graph embedding in VAE. This avoids being confined to a single point in the latent space. If the input is $X$ and the parameters of the multivariate normal distribution are $Z$, then in the encoder, the probability of $Z$ given $X$ is denoted as $p\left(Z\mid X\right)$, and in the decoder, the probability of $X$ given $Z$ is denoted as $q\left(X\mid Z\right)$. In the encoder, the input $X$ is known, and the variable is $Z$, while in the decoder, the parameters of the multivariate normal distribution $Z$ are known. VGAE aims to make $p\left(Z\mid X\right)$as close as possible to $q\left(X\mid Z\right)$. $p\left(Z\mid X\right)$. In the VGAE framework, the encoder is responsible for determining the probability distribution of the latent variable $Z$ given the input $X$. The goal is to capture the latent representation that best represents the input data. The subsequent steps in VGAE involve the decoder and the optimization process to align the distributions $p\left(Z\mid X\right)$ and $q\left(X\mid Z\right)$ for effective graph reconstruction and generation. To achieve this, the process begins with the encoder: Please note that there are two encoders here. GCN1 is used to generate a low-dimensional embedding vector $\overline{X}= GCN1\left(A,X\right)= ReLU\left(\tilde{A}X{W}_0\right)$, GCN2 is used to generate $\log{\sigma}^2$ and $\mu$，where $\log{\sigma}^2= GCN{2}_{\sigma}\left(X,A\right)=\tilde{A}\overline{X}{W}_1$ and $\mu = GCN{2}_{\mu}\left(X,A\right)=\tilde{A}\overline{X}{W}_1$(Figure 1D, b).

Therefore, the GCN layer is represented as:


(7) \begin{equation*} GCN\left(A,X\right)=\tilde{A} ReLU\left(\tilde{A}X{W}_0\right){W}_1 \end{equation*}

Next is the use of reparameterization (Figure 1D, c):


$$ Z=\mu +\sigma \odot \epsilon $$

(8) \begin{equation*} \epsilon \sim Norm\left(0,1\right) \end{equation*}

Finally, the decoder part (Figure 1D, d):


(9) \begin{equation*} \hat{A}=\mathrm{logistic}\ \mathrm{sigmoid}\left(z{z}^T\right) \end{equation*}

In AVGN, the preprocessed input features X undergo encoding through the encoder (which includes the MHA block), resulting in a latent representation of dimension 20. This representation is then concatenated with the re-parameterization output, yielding an embedding *Z* of dimension 28. Finally, the decoder is employed to reconstruct features with a dimension of 128.

### Performance evaluation

In this manuscript, we employ the Adjusted Rand Index (ARI) as a key evaluation metric to measure the similarity between two partitions. It calculates the number of data points assigned the same or different labels in the two partitions, providing a quantitative assessment of their resemblance.


(10) \begin{equation*} ARI=\frac{\sum_{ij}\kern0.1em \left(\genfrac{}{}{0pt}{}{n_{ij}}{2}\right)-\left[\sum_i\kern0.1em \left(\genfrac{}{}{0pt}{}{a_i}{2}\right)\cdotp \sum_j\kern0.1em \left(\genfrac{}{}{0pt}{}{b_j}{2}\right)\right]/\left(\genfrac{}{}{0pt}{}{n}{2}\right)}{\frac{1}{2}\left[\sum_i\kern0.1em \left(\genfrac{}{}{0pt}{}{a_j}{2}\right)+\sum_j\kern0.1em \left(\genfrac{}{}{0pt}{}{b_j}{2}\right)\right]-\left[\sum_i\kern0.1em \left(\genfrac{}{}{0pt}{}{a_j}{2}\right)\cdotp \sum_j\kern0.1em \left(\genfrac{}{}{0pt}{}{b_j}{2}\right)\right]/\left(\genfrac{}{}{0pt}{}{n}{2}\right)} \end{equation*}

Differing from Normalized Mutual Information (NMI), ARI takes into account the influence of random assignments, ensuring a more precise outcome. ARI values span from −1 to 1. Positive values signify superior clustering performance compared to random assignment, while negative values indicate subpar clustering results in contrast to random assignment. A value of 0 suggests similarity to random assignment.

NMI quantifies the degree of information shared between the clustering result and the reference partition. NMI values generally fall within the range of 0 to 1, where increased values signify greater similarity between the clustering outcome and the reference partition. NMI is less sensitive to the size of partitions and the number of categories, making it robust when dealing with different partition sizes and category numbers.


(11) \begin{equation*} NMI\left(X;Y\right)=2\frac{I\left(X;Y\right)}{H(X)+H(Y)} \end{equation*}

## RESULTS

In order to comprehensively illustrate the performance, versatility and potential applications of AttentionVGAE across various datasets, we designed and conducted experimental work.

### Overview of AttentionVGAE workflow

Firstly, AttentionVGAE integrates and reduces the dimensionality of histology, gene expression and spatial location information (Figure 1A and B), utilizing a pre-trained MaxVit model for learning image features (Figure 1D, a). Subsequently, this integrated information is input into a VGAE guided by a multi-head attention mechanism (Figure 1C). The latent embeddings obtained from the encoder are employed to construct a cell adjacency network, which undergoes Leiden clustering. Detailed explanations for this section can be found in the VGAE component of the Materials and Methods section in this manuscript. Following that, refinement is applied to enhance the clustering results. Finally, the clustered results are visualized in the spatial domain by incorporating spatial location information (Figure 1D).

### AttentionVGAE underwent benchmark testing on the DLPFC dataset

To evaluate the performance difference of AttentionVGAE relative to state-of-the-art techniques in spatial domain detection, we conducted a comparative analysis involving AttentionVGAE and seven other detection methods: Scanpy, stLearn, SEDR, SpaGCN, BayesSpace, DeepST and STAGATE. It’s worth noting that Leiden, implemented in Scanpy, is categorized as a non-spatial clustering method, while the others belong to spatial clustering methods.

**Figure 2:**
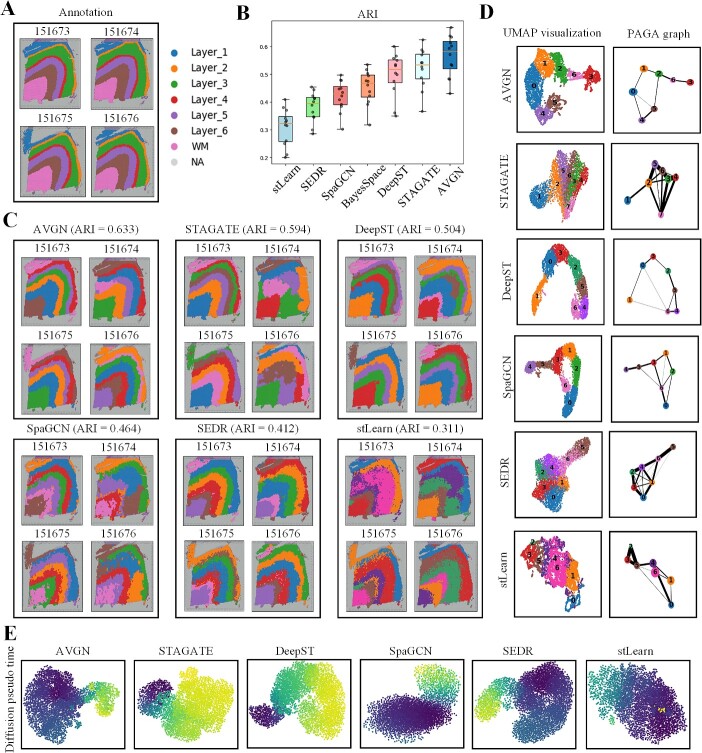
The benchmark test results of AttentionVGAE compared to state-of-the-art spatial domain detection methods on the DLPFC dataset. (**A**) presents the ground truth benchmark of 151 673 samples manually annotated in the DLPFC dataset. (**B**) exhibits boxplots that compare the ARI metrics among different spatial domain detection methods applied to the DLPFC dataset. Each detection method underwent 12 tests. (**C**) showcases the spatial domain detection results of AttentionVGAE corresponding to ARI = 0.633, along with visualizations of the UMAP [51] and PAGA graphs [52]. (**D**) exhibits the test results of five selected other state-of-the-art spatial domain detection methods (choosing the highest ARI among the 12 tests) and their corresponding visualizations of UMAP and PAGA graphs.

In the Uniform Manifold Approximation and Projection (UMAP) visualizations, noteworthy observations come to light. The clustering results of stLearn reveal a dissonant and indistinct pattern in Figure 2D, indicating a lack of clearly defined hierarchical contours within the spatial domain visualization (Figure 2C). SEDR, SpaGCN and BayesSpace exhibit relatively clear hierarchical boundaries, but there are more outliers near the edges of the layers, leading to a sense of discontinuity in the layers (Figure 2C). DeepST shows significant deficiencies in defining the boundaries of the overall smaller cortical layer [53] and larger cortical layer. STAGATE improves on this issue, particularly evident in the visualizations of Layer_0 to Layer_4 (Figure 2C). In the visualizations of AttentionVGAE’s detection results, the clustering performance of Layer_2, Layer_3 and Layer_5 is notably superior to other spatial clustering methods. The edge of the clustering visualization results is more accurate, clear and smooth, demonstrating excellent clustering performance (Figure 2C). In the UMAP visualizations of the 151 673 samples, data processed by AttentionVGAE exhibit outstanding results in the scatter plot. The clustering points of the seven cortical layers are more clearly concentrated. Compared to the results of other detection methods, AttentionVGAE’s UMAP results have fewer outliers. This indicates that AttentionVGAE has a stronger ability to perceive structural heterogeneity [54]. Lastly, a temporal analysis visualization of the six key detection methods tested in this manuscript for the 151 673 samples reveals their differentiation trajectories, which is crucial for studying cell differentiation trajectories (from dark colors to light colors) (Figure 2E). Notably, AttentionVGAE’s average ARI is significantly higher than other spatial domain detection methods, with an average ARI of 0.592 (Figure 2B).

### AttentionVGAE demonstrates exceptional capabilities in detecting fine structures on coronal sections of the adult mouse brain (fresh-frozen)

**Figure 3:**
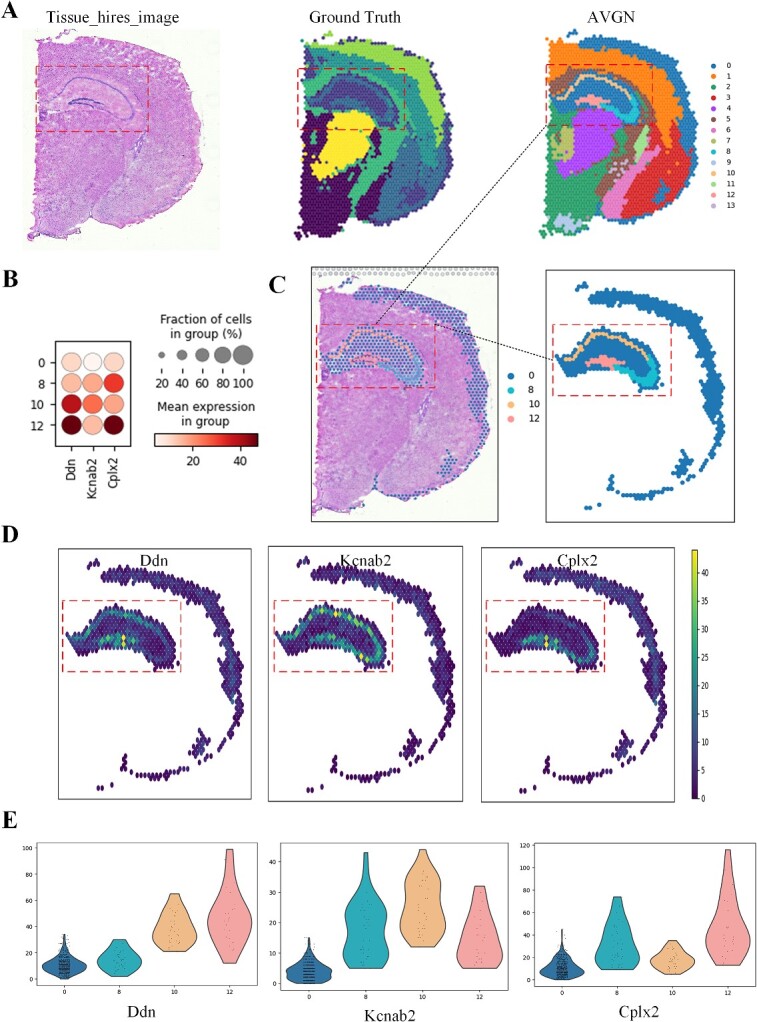
The spatial domain detection conducted by AttentionVGAE on coronal sections of the adult mouse brain (fresh-frozen). (**A**) includes a high-resolution tissue image of the coronal section of the adult mouse brain, the ground truth of spatial domain detection for adult mouse samples and the visualization of AttentionVGAE’s spatial domain detection results on experimental data. (**B**) displays the average expression levels of genes Ddn, Kcnab2 and Cplx2 in Clusters 0, 8, 10 and 12. These three genes were selected through differential gene analysis as highly variable genes. The proportion of cells containing these genes in the total cell population of the corresponding cell clusters is also shown. (**C**) provides detailed views of fine structures in the spatial domain detection results. (**D**) shows the spatial distribution maps of genes Ddn, Kcnab2 and Cplx2 at fine structure locations. (**E**) includes violin plots of gene expression levels for Ddn, Kcnab2 and Cplx2 in the clusters.

In the spatial domain visualization of AttentionVGAE’s results in detecting fine structure positions from (Figure 3C), clear and distinct boundaries can be observed, forming an overall pattern that is both elegant and smooth. Upon consulting the average expression levels of highly variable genes and the proportion of cells harboring these genes in Figure 3B, a discernible correlation emerges, showcasing the intricate relationship between the finely identified spatial positions by AttentionVGAE and the expression patterns of differentially expressed genes. This correlation is visually substantiated through the depictions in Figure 3D and E. Based on these results, the incorporation of the multi-head attention mechanism in GVAE directs the focus toward essential aspects of the data, facilitating fine-grained modeling. In clustering tasks, this implies that the model can better distinguish between different categories or clusters and more accurately capture the inherent structure of the data. Therefore, AttentionVGAE demonstrates effective capabilities in spatial domain detection of data with fine structure positions.

### AttentionVGAE accurately performs spatial domain detection on adult human glioblastoma multiforme

**Figure 4:**
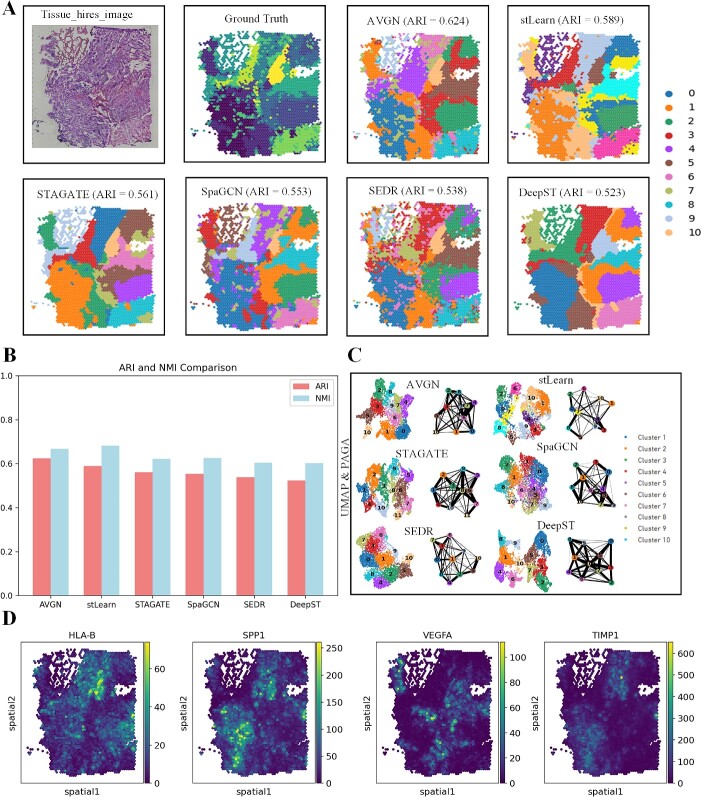
The comparative results of AttentionVGAE and the latest spatial domain detection methods on the GBM dataset. (**A**) includes a high-resolution tissue image of GBM, the ground truth of spatial domain detection for GBM and visualizations of spatial domain detection results for six methods. (**B**) presents bar charts depicting the ARI and NMI metrics for the spatial domain detection results of the six methods. (**C**) showcases UMAP plots and PAGA analysis graphs for the detection results of the six spatial domain detection methods. (**D**) displays spatial visualizations of four differentially expressed genes.

From (Figure 4A), it can be observed that AttentionVGAE’s detection results exhibit the best average ARI performance. However, by examining the NMI metric, it is noted that AttentionVGAE’s NMI is slightly lower than the results from stLearn (Figure 4B). Employing both NMI and ARI contributes to a more comprehensive clustering evaluation, leveraging their distinct advantages in handling partitions of varying sizes and different numbers of categories. NMI demonstrates robustness in dealing with partitions of different sizes and category numbers, while ARI considers the impact of random assignment, potentially offering more accurate results in certain situations. The combined use of these two metrics allows for a more comprehensive assessment of clustering algorithm performance, reducing subjective bias. Careful examination of the UMAP plots in Figure 4C reveals that AttentionVGAE’s detection results also exhibit superior performance.

Analyzing the spatial domain detection results of AttentionVGAE concerning highly variable differentially expressed genes within the tumor [55], we identified several genes contributing to the heterogeneity of tumor tissue. In this manuscript, we focused on the spatial visualization of gene expression for HLA-B [56], SPP-1 [57], VEGF-A [58] and TIMP-1 [59, 60]. In the spatial domain detection visualization results of AttentionVGAE, significant expression of HLA-B is observed in regions 2, 3, 5 and 10 (Figure 4D-1). HLA-B is a human leukocyte antigen involved in immune system recognition and antigen presentation. It assumes a vital function in immune responses, specifically in identifying and eradicating infections or aberrant cells. Elevated expression of HLA-B may contribute to enhancing the immune system’s recognition and attack against GBM cells, potentially exerting a positive effect on inhibiting tumor growth.

Differential expression of SPP-1 is distributed in regions 0, 4, 5 and 7 (Figure 4D-2). SPP-1, a secreted phosphoprotein, participates in various biological processes, including cell adhesion, migration, invasion and inflammation. In tumors, elevated expression of SPP-1 is associated with processes such as tumor growth, invasion and angiogenesis. High expression of SPP-1 in GBM may be linked to increased invasiveness and poor prognosis. It may influence disease progression by promoting tumor cell migration and invasion and regulating the tumor microenvironment. VEGF-A, a crucial factor in angiogenesis, shows heightened expression in regions 1 and 3 (Figure 4D-3).

In tumors, elevated levels of VEGF-A are typically associated with neoangiogenesis (angiogenesis). In GBM, high expression of VEGF-A may promote the formation of blood vessels around the tumor, providing the necessary oxygen and nutrients for tumor growth. This may contribute to tumor growth and invasion and may be associated with poor prognosis. On the other hand, different levels of TIMP-1 are also distributed in regions 0, 6 and 10 (Figure 4D-4). In GBM, the expression level of TIMP-1 may affect the activity of MMPs, thereby regulating cell migration and invasion. Elevated levels of TIMP-1 could be linked to heightened tumor invasiveness and an unfavorable prognosis.

These findings highlight AttentionVGAE’s potential to enhance our biological understanding by delving into the potential heterogeneity within the tumor. This is apparent from both spatial domain detection and gene expression patterns. The attention-guided mechanism provides VGAE with enhanced flexibility, enabling adaptive focus on various features or relationships within the data. This adaptive capability allows the model to better capture potential patterns based on the actual structure of the data.

### AttentionVGAE further reveals heterogeneity in infiltrating ductal carcinoma data

**Figure 5:**
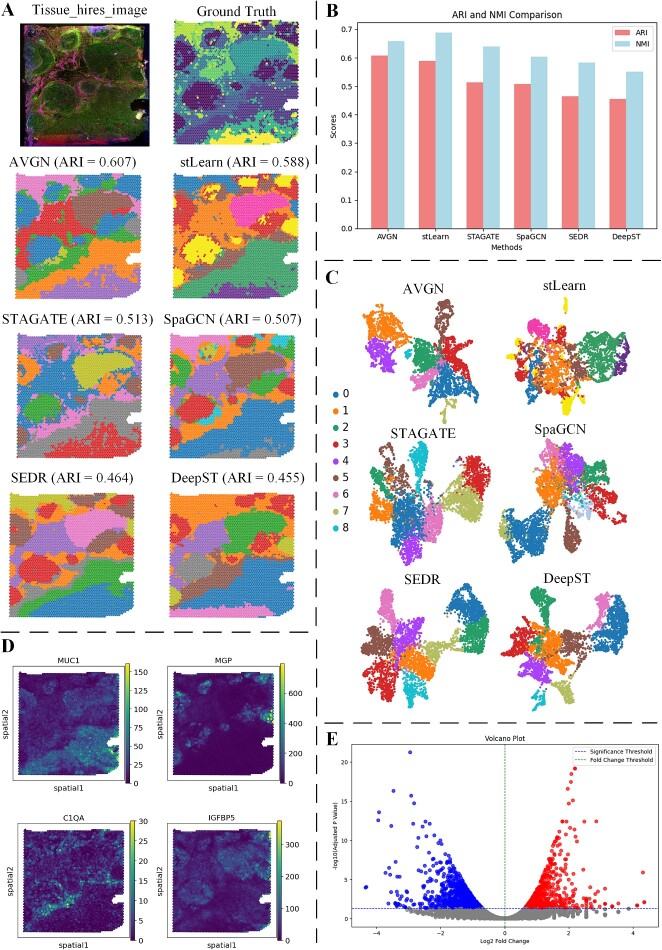
Compares the spatial domain detection results of AttentionVGAE with the latest methods on the infiltrating ductal carcinoma (IDC) dataset. (**A**) includes high-resolution tissue images of IDC, the ground truth of spatial domain detection for IDC and visualizations of spatial domain detection results for six methods. (**B**) presents bar charts depicting the ARI and NMI metrics for the spatial domain detection results of the six methods. (**C**) Showcases UMAP plots for the detection results of the six spatial domain detection methods. (**D**) Displays spatial expression visualization of genes specific to differential expression analysis. (**E**) Visualizes the results of differential expression analysis in a volcano plot.

Through Figure 5A and B, we observed that AttentionVGAE’s detection results have the highest ARI but a slightly lower NMI compared to stLearn’s performance. This situation, rarely encountered in previous research and other articles, suggests the importance of considering multiple metrics for a comprehensive and rigorous analysis. stLearn has historically exhibited poorer performance in recent studies, including our comparative experiments on the DLPFC dataset (Figure 2C). However, in this study of tumor and cancer cell datasets, stLearn exhibited robust performance, highlighting its potential for spatial domain detection in these datasets.

Additionally, in this manuscript, we investigated four differentially expressed genes: MUC1 [61], MGP [62], C1QA [63] and IGFBP5 [64] (Figure 5D). MUC1 encodes an adhesion protein that typically forms a protective mucous layer on the surface of epithelial cells. In cancer, abnormal expression of MUC1 is associated with cell adhesion, proliferation and invasion. MUC1 is often overexpressed in breast cancer, correlating with cancer cell infiltration and migration. MGP encodes a vitamin K-dependent protein involved in calcium metabolism in bone and soft tissue. It is also thought to contribute to the progression of tumors. In the latest research, MGP’s role in infiltrating ductal carcinoma (IDC) has yet to be fully explored, but it is related to its potential role in cell adhesion, proliferation and migration. C1QA encodes a C1q protein subunit in the immune system, participating in the formation of immune complexes. The specific role of C1QA in IDC may be related to the interaction between the immune system and the tumor microenvironment. IGFBP5 encodes an insulin-like growth factor-binding protein that regulates cell growth and apoptosis. IGFBP5 may play a regulatory role in cell growth and invasion in breast cancer. Analysis through AttentionVGAE provides essential evidence for further studying the impact of differentially expressed genes in infiltrating ductal carcinoma on specific regions. It contributes to understanding the heterogeneity of gene expression in spatial terms, subsequent clinical analyses and research on potential prognostic risk genes.

AttentionVGAE excels in UMAP analysis (Figure 5C), indicating that the AttentionVGAE method with an added attention mechanism is more adept at capturing the intrinsic structure and relationships within the data for this specific task. In the presence of complex features and patterns, a model needs adaptability and flexibility, attributes provided by the attention mechanism. The success of AttentionVGAE may result from its ability to dynamically focus on crucial features or relationships within the data, showcasing superior handling of heterogeneity and noise. This capability enhances the accuracy of clustering and spatial domain detection.

### AttentionVGAE (adaptive) performs spatial domain detection on high- spatial-resolution samples from the Stereo-seq and Slide-seq platforms

**Figure 6:**
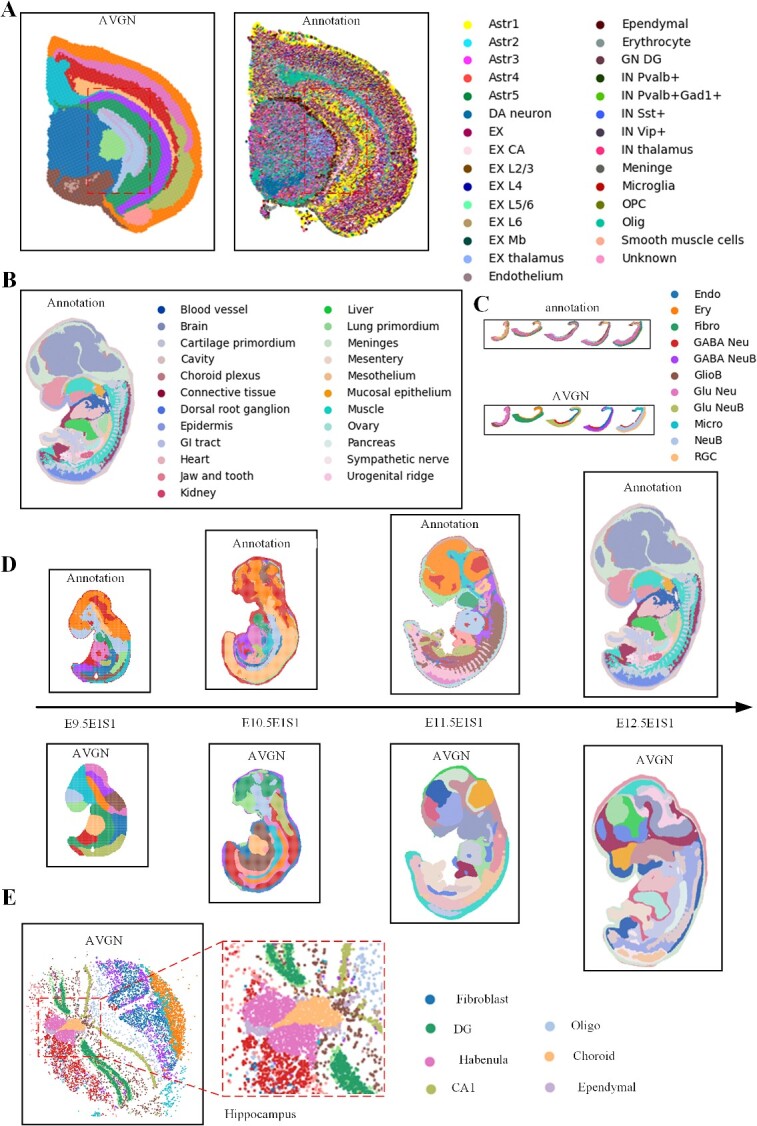
AttentionVGAE performs spatial domain detection with adaptive clustering on high=spatial-resolution samples from the Stereo-seq and Slide-seq platforms. (**A**) AttentionVGAE (adaptive) detects spatial domain information in the adult mouse hemi-brain data from the Stereo-seq platform. (**B**) Annotation of sample E12.5E1S1. (**C**) AttentionVGAE (adaptive) detects spatial domain information in the Dorsal_midbrain_cell sample from the Stereo-seq platform. (**D**) AttentionVGAE (adaptive) demonstrates spatial domain detection performance on mouse embryo section data. (**E**) AttentionVGAE (adaptive) identifies intricate structures in the Slide-seq Hippocampus dataset.

The spatial position of cells in tissues strongly influences their function, but the lack of high-throughput, whole-genome gene expression readings with cellular resolution is a challenge. The Slide-seq technique’s high spatial resolution is crucial for mapping cellular atlases. Aggregating data into larger feature sizes poses challenges in resolving cell types within heterogeneous tissue regions. AttentionVGAE effectively detects fine-scale structures in mouse brain data from the Stereo-seq and Slide-seq platforms. In the data from Stereo-seq’s adult mouse hemi-brain sections, AttentionVGAE with adaptive clustering not only detects fine-scale structures clearly but also filters out noise to a large extent (Figure 6A). When performing spatial domain detection on Hippocampus data from the Slide-seq platform, AttentionVGAE’s results are highly consistent with the Slide-seq platform, especially in capturing fine-scale structures (Figure 6E).

AttentionVGAE aims to conduct spatial domain detection by utilizing continuous data from mouse embryo section data obtained from the Stereo-seq platform, revealing the dynamic differentiation process of developmental structures. The results indicate that AttentionVGAE (adaptive) significantly identifies some continuous differentiation processes in the embryo (Figure 6D). Furthermore, AttentionVGAE (adaptive) also demonstrates considerable spatial domain detection capabilities on high-spatial-resolution datasets (Figure 6E). This is closely associated with the excellent performance of the multi-head attention mechanism in handling high-dimensional data, enabling the model to simultaneously focus on multiple aspects of the data, thereby enhancing its ability to capture both local and global relationships in the data. Consequently, AttentionVGAE achieves remarkable spatial domain detection capabilities for high-spatial-resolution datasets.

### Performing ablation experiments on AttentionVGAE

The purpose of this section of ablation experiments is to further explore the contribution of the MAH block to the overall AVGN method. Conducting a more in-depth analysis will shed light on the value information of VGAE and the attention module in spatial domain recognition.

**Figure 7:**
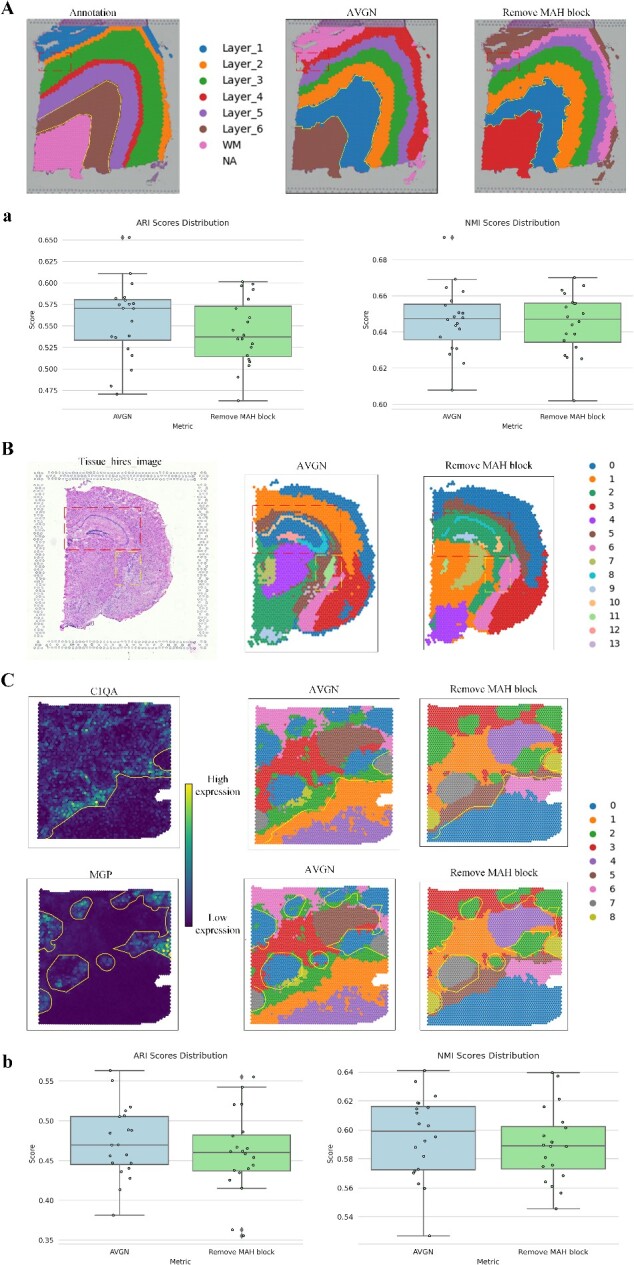
Results of ablation experiments on three datasets. (**A**) results of ablation experiments on the DLPFC dataset (151 673 slices) are displayed. From left to right: true labels, spatial domain detection results using AVGN and spatial domain detection results after removing the MHA block from AVGN. The artificially marked dashed boxes and dashed outlines are included for reference. (**B**) results of spatial domain detection on adult mouse brain slices are presented, emphasizing fine-grained comparisons of locations indicated by dashed boxes. (**C**) shows the results of ablation experiments focusing on the heterogeneity of infiltrating ductal carcinoma. The upper left and lower left display expression distributions of C1QA and MGP genes, respectively, along with dashed lines indicating manually annotated regions of gene heterogeneity. The four images on the right demonstrate the comparative effects of ablation experiments, also marked with the same dashed lines. (**a**) The results of the ablation experiment were identified and quantified for 20 times on the sample data of 151 673. On the left is the line box diagram of ARI score, and on the right is the NMI score. (**b**) Quantitative results of 20 spatial domain identification ablation experiments were performed on invasive ductal carcinoma data. On the left is a wireframing of the ARI score and on the right is the NMI score.

In the comparative experiment on the 151 673 slices of the DLPFC, the selected result represents the network outcome with the highest ARI value among 10 runs after removing the MAH block (Figure 7A, right), ARI = 0.582. It can be observed that, for the results after removing the MAH block, AVGN exhibits a more excellent performance in spatial domain detection for Layer_2 in the region marked by the red dashed box, while subtle fractures appear in the results after removing the module. After observing the boundary lines between Layer_6 in the detection results, it is evident that AVGN with the MAH block has smoother and more continuous lines for inter-layer boundaries. We believe this is closely related to the attention mechanism’s ability to represent spatial domain boundaries. After conducting 20 comparative experiments, it was observed that AVGN achieved higher ARI and NMI scores compared to the models without the MHA block (Figure 7C, a).

To explore the value of VGAE and the attention module in the spatial domain detection of fine positions, we chose the adult mouse hippocampus as the spatial domain detection object for ablation experiments. In Figure 7B (right), the spatial domain of the mouse hippocampus (highlighted by the border) shows obvious fractures in the results after removing the MAH block, while AVGN detects only extremely subtle fractures, providing an overall coherent and complete detection of the hippocampus. Additionally, the part within the dashed box clearly demonstrates AVGN’s higher sensitivity to structural heterogeneity compared to the network without the MAH block.

Finally, we attempted to reveal the impact of the attention mechanism on capturing gene expression differences in infiltrating ductal carcinoma data (Figure 7C). Through the experiment, we found that the attention mechanism has a minor impact on the general area of gene expression. However, upon closer observation of the region marked by the line, we observed that attention mechanism appear to have a compacting effect on spatial domain detection results for locally high gene expression. This effect is faintly evident in the marked area of the control group for the MGP gene. In the results of the C1QA gene control group, we found that AVGN detected more details compared to the network without the MAH block. After conducting 20 comparative experiments, it was observed that both ARI and NMI scores improved when the MHA block was added to AVGN (Figure 7C, b).

## DISCUSSION

As deep learning technology progresses, attention mechanisms and GNNs are increasingly prevalent in spatial transcriptomics research. Our contribution, AttentionVGAE, innovatively integrates these advancements into a variational graph neural network encoder, guided by attention mechanisms. This unique combination allows for adaptive focus on different nodes or edge information within the graph, enhancing model performance, particularly in scenarios lacking prior knowledge of cluster quantity.

One key innovation of AttentionVGAE lies in its ability to address the calibration of low-quality gene expression data and balance attention between local and global structures. This dual focus ensures accurate representation of gene expression patterns while capturing fine-grained local variations and broader tissue structural relationships.

In rigorous benchmark testing, AttentionVGAE demonstrated exceptional performance across various tasks, including detecting fine structures in coronal sections of the adult mouse brain, identifying spatial domains in human glioblastoma multiforme and infiltrating ductal carcinoma datasets and conducting spatial domain detection on high-spatial-resolution samples from platforms like Stereo-seq and Slide-seq. These results underscore the practical utility of AttentionVGAE in advancing spatial transcriptomics research.

Overall, AttentionVGAE has made progress in spatial domain recognition by correcting low-quality gene expression, utilizing multi-head attention modules, graph convolutional encoders and graph deconvolution decoders. However, due to the sparsity of SRT data, AttentionVGAE still faces limitations in correcting low-quality gene expression. Therefore, the next critical step should involve considering the integration of single-cell sequencing data, which offers single-cell resolution, to further calibrate SRT data with high-resolution data. This advancement can drive improvements in the performance of tissue spatial domain recognition.

**Key Points**

- AttentionVGAE is a novel approach in spatial transcriptomics research, adept at capturing spatial domain information while addressing challenges like low-quality gene expression calibration and balancing attention between local and global structures.
- Our architecture integrates multi-head attention with variational graph autoencoder, offering an adaptive solution for scenarios lacking prior cluster quantity knowledge.
- Leveraging a graph convolutional neural network framework, AttentionVGAE precisely models tissue microenvironment structural relationships using histological images, genetic data and spatial positions.
- Test results show AttentionVGAE’s superiority in spatial domain detection, advancing our understanding of tissue gene expression patterns and genomic research.

## Data Availability

The paper utilizes publicly available datasets for its analysis. These include the following: (1) Human DLPFCs within the spatialLIBD at http://spatial.libd.org/spatialLIBD. (2) Adult mouse brain (fresh-frozen), Adult Human Glioblastoma Multiforme and Infiltrating ductal carcinoma datasets at https://support.10xgenomics.com/spatial-gene-expression/datasets. (3) Mouse embryo data, Dorsal_midbrain_celand and Adult mouse hemi-brain data at https://db.cngb.org/stomics/mosta. (4) Hippocampus dataset at https://portals.broadinstitute.org/single_cell/study/slide-seq-study.

## CODE AVAILABILITY

The code for AVGN can be obtained at https://github.com/Listen-lei/AttentionVGAE-main.

## Author Biographies

**Lixin Lei** is a graduate student at the Academy of Artificial Intelligence, Beijing Institute of Petrochemical Technology. His expertise is bioinformatics and machine learning.

**Kaitai Han** is a graduate student at the Academy of Artificial Intelligence, Beijing Institute of Petrochemical Technology. His expertise is deep learning.

**Zijun Wang** is a graduate student at the Academy of Artificial Intelligence, Beijing Institute of Petrochemical Technology. His expertise is deep learning.

**Chaojing Shi** is a graduate student at the Academy of Artificial Intelligence, Beijing Institute of Petrochemical Technology. His expertise is deep learning.

**Zhenghui Wang** is a graduate student at the Academy of Artificial Intelligence, Beijing Institute of Petrochemical Technology. Her expertise is bioinformatics.

**Ruoyan Dai** is a graduate student at the Academy of Artificial Intelligence, Beijing Institute of Petrochemical Technology. Her expertise is bioinformatics.

**Zhiwei Zhang** is a graduate student at the Academy of Artificial Intelligence, Beijing Institute of Petrochemical Technology. His expertise is deep learning.

**Mengqiu Wang** is a graduate student at the Academy of Artificial Intelligence, Beijing Institute of Petrochemical Technology. Her expertise is bioinformatics.

**Qianjin Guo** is a professor at the Academy of Artificial Intelligence, Beijing University of Petrochemical Technology. His main research fields include intelligent equipment and systems, machine learning, biomedical imaging and ultrafast spectroscopy.
